# Elastase and Cathepsin G from Primed Leukocytes Cleave Vascular Endothelial Cadherin in Hemodialysis Patients

**DOI:** 10.1155/2014/459640

**Published:** 2014-05-04

**Authors:** Meital Cohen-Mazor, Rafi Mazor, Batya Kristal, Shifra Sela

**Affiliations:** ^1^Bruce Rappaport School of Medicine, Technion, 31096 Haifa, Israel; ^2^Eliachar Research Laboratory, Western Galilee Hospital, 22100 Naharyia, Israel; ^3^Nephrology Unit, Western Galilee Hospital, 22100 Nahariya, Israel; ^4^Faculty of Medicine in the Galilee, Bar Ilan University, 13100 Safed, Israel

## Abstract

*Aims*. To test the hypothesis that primed PMNLs in blood of chronic kidney disease patients release the active form of elastase and cathepsin G causing degradation of vital proteins and promote tissue damage. * Methods*. RT-PCR, immunocytochemical staining, immunoblotting, and FACS analyses were used to study these enzymes in hemodialysis patients (HD) versus healthy normal controls (NC). Using PMNLs and endothelial cells cocultivation system we measure the effect of HD PMNLs on the endothelial VE-cadherin, an essential protein for maintaining endothelial integrity. * Results*. Levels of elastase and cathepsin G were reduced in PMNLs of HD patients, while mRNA enzymes levels were not different. Elevated levels of the active form of these enzymes were found in blood of HD patients compared to NC.HD plasma had higher levels of soluble VE-cadherin present in three molecular forms: whole 140 kDa molecule and two fragments of 100 and 40 kDa. Cocultivation studies showed that primed PMNLs cleave the endothelial cadherin, resulting in a 100 kDa fragment. * Conclusions*. Elastase and cathepsin G are elevated in the plasma of HD patients, originating from primed PMNLs. In these patients, chronic elevation of these enzymes contributes to cleavage of VE-cadherin and possible disruption of endothelial integrity.

## 1. Introduction

Elastase and cathepsin G are serine proteinases contained in the azurophilic granules of peripheral polymorphonuclear leukocytes (PMNLs) and degrade proteins of phagocytosed microorganisms [1, 2]. Stored after synthesis in their active form, these proteolytic enzymes have potential deleterious effects. When released, they act on substrates comprising the connective tissue of the lung, blood vessels, joints, and other organs, causing tissue destruction [3–9]. Circulating proteinase inhibitors, such as serum proteins *α*1-antitrypsin (*α*1-AT) and *α*1-antichymotrypsin (*α*1-ACT), neutralize the activity of these proteinases in plasma [10, 11]. The clinical significance of elastase and cathepsin G is most evident under conditions in which an imbalance develops between these enzymes and their inhibitors, enabling the circulating active enzymes to induce vascular injury.

PMNL serine proteinases have been implicated in the pathogenesis of diseases associated with PMNL priming such as rheumatoid arthritis [12, 13]. PMNL priming is also associated with pathological disorders associated with atherosclerosis and cardiovascular diseases such as hypertension, diabetes, and end stage renal disease treated with chronic hemodialysis (HD) [14–16]. Studies have shown that purified elastase and cathepsin G can cleave the extracellular part of vascular endothelial (VE) cadherin, an essential protein for maintaining vascular endothelial integrity [17, 18], and that elastase and cathepsin G bound to their inhibitor are released during hemodialysis sessions [19].

In this study, we sought out to test the hypothesis that even before dialysis HD PMNLs are primed, thus contributing to elevated levels of elastase and cathepsin G in the plasma. We have also evaluated cleavage of VE-cadherin mediated by these proteinases found in HD plasma.

Our findings consist of elevated levels of these proteins in HD plasma. Plasma of HD had higher levels of VE-cadherin fragments, the result of this molecule cleavage by active elastase and cathepsin G. HD PMNLs as well as purified enzymes cleaved VE-cadherin from cultured endothelial cells. Taken together, this study provides a novel mechanism by which active elastase and cathepsin G originating from primed PMNLs of HD patients can initiate endothelial dysfunction.

## 2. Materials and Methods

### 2.1. Patients and Blood Samples

Ten hemodialysis patients and healthy control subjects were enrolled in this study (Table 1). Blood for the determination of biochemical and hematological parameters and for the isolation of PMNLs was drawn into citrate tubes, and in HD patients from the arterial line immediately before a dialysis session. All patients underwent hemodialysis three times a week; each dialysis treatment lasted 4 hours and was carried out with low flux polysulfone membranes (F8, Fresenius Medical Care, Bad Homburg, Germany). The water for dialysis met the standards of the Association for the Advancement of Medical Instrumentation (AAMI). Patients with evidence of acute or chronic infection, malignancy, or who had received a blood transfusion within three months prior to blood sampling were excluded. All participants signed an informed consent for blood sampling, and the study was approved by the Institutional Committee in accordance with the Helsinki declaration.

### 2.2. PMNL Isolation and Analysis

PMNLs were isolated as described previously [15]. Isolated PMNLs (>98% pure, approximately 10^7^ cells per isolation) were resuspended and counted in phosphate buffered saline (PBS, Beit Haemek, Israel) containing 0.1% glucose. PMNL priming was assessed by the rate of superoxide release [14], and by the surface levels of CD11b, as described previously [20]. The rate of superoxide release was determined after cell stimulation with 0.32 × 10^−7^ M phorbol 12-myristate 13-acetate (PMA, Sigma, St. Louis, MO). The assay is based upon superoxide dismutase (SOD) inhibitable reduction of 80 *μ*M cytochrome C (Sigma, St. Louis, MO) to its ferrous form. The change in optical density was monitored at 549 nm, as described previously [14]. Expression of CD11b on PMNLs in whole blood was determined using the FC500 flow cytometer (Beckman-Coulter). Fifty *μ*L of blood was incubated for 10 min with anti CD11b-PE conjugated monoclonal antibody (IQ Products, The Netherlands), followed by RBC lysis (Q-prep method; Coulter Corporation, Hialeah, FL, USA). To enable gating on the PMNL population, anti CD16, conjugated to PC5 monoclonal antibody (Immunotech, Marseille, France), was used. Surface levels of CD11b on PMNLs are expressed as mean fluorescence intensity (MFI), after subtracting the nonspecific background.

### 2.3. RNA Isolation and RT-PCR of Elastase and Cathepsin G

RNA was isolated using Tri Reagent according to the manufacturer's (Sigma) instructions. Complementary DNA was synthesized according to Krug and Berger [21], using an OmniGene thermal cycler (Hybaid, UK). The resulting cDNAs were amplified by PCR using 2 *μ*L aliquots of RT reaction incubated with 0.2 mM dNTPs, 2 *μ*L of Taq DNA polymerase buffer, 0.6 U of TaqDNA polymerase (BioLine, UK), and 10 pmoles of the specific primers. The oligonucleotide sequence for elastase, according to Hirata et al. [22], is sense (5′-AGTGCCTGGCCATGGGCTGG-3′) and antisense (5′-CACCGGGGCAAAGGCATCGG-3′) with a final PCR product of 257 bp. The PCR conditions were initial denaturation at 94°C for 5 minutes, 30 PCR cycles of denaturation at 94°C for 1 minute, annealing at 55°C for 2 minutes, and extension at 72°C for 3 minutes. The oligonucleotide sequence for cathepsin G, according to Hirata et al. [22], is sense (5′-TGAGAGTGCAGAGGGATAGG-3′) and antisense (5′-CAGGAAACTTGAGACCCTGC-3′) with a final PCR product of 216 bp. The PCR conditions were identical to those described above for elastase. Aliquots (15 *μ*L) of the amplified cDNA of elastase and cathepsin G were separated on 1% agarose gel electrophoresis, visualized by ethidium bromide staining, and compared to a housekeeping gene, *β*-actin. The 200 bp product of actin was amplified using the following primers: sense (5′-CCTTCCTGGGCATGGAGTCCTG-3′), and antisense (5′-GAGCAATGATCTTGATCTTC-3′). The densities of the enzyme PCR products were calculated in each gel relative to the actin product.

### 2.4. Detection and Quantificationof Elastase and Cathepsin G in PMNLs

*(1) Intracellular Levels of Elastase and Cathepsin G in PMNLs*. The levels of intracellular elastase and cathepsin G were measured in whole blood by flow cytometry using polyclonal rabbit anti-human elastase and anti-human cathepsin G antibodies (United States Biological, Massachusetts, USA), followed by a secondary FITC-conjugated fluorescent anti-rabbit antibody (Chemicon International, CA). PMNLs were permeabilized with the FIX & PERM cell permeabilization kit. Anti CD16- PC5 (Immunotech, Marseille, France) was used for gating on PMNLs. Total rabbit IgG antibodies (United States Biological, Massachusetts, USA), followed by a secondary FITC-conjugated fluorescent anti-rabbit antibody, served as nonspecific controls of the fluorochrome. The results are presented as MFI per cell after subtracting the nonspecific background.

*(2) Localization of Elastase and Cathepsin G in PMNLs*. Human PMNLs were stained by indirect immunohistochemical staining. Whole blood slides were prepared by fixation and permeabilization with methanol (−20°C, 3 minutes). The slides were stained with specific anti-elastase and anti-cathepsin G antibodies (United States Biological, Massachusetts, USA). The Histofine Simple Stain kit (Nichirei Corporation, Japan) was used for immunohistochemical staining of PMNLs according to the manufacturer's instructions.

### 2.5. Detection and Quantification of Elastase and Cathepsin G in Plasma

Plasma of NC subjects and HD patients was depleted of albumin and immunoglobulins by ProteoPrep Blue Albumin Depletion Kit (PROT-BA KIT) (Sigma-Aldrich, USA). Following the addition of Laemli loading buffer containing 3% *β*-mercaptoethanol, the plasma was boiled at 100°C for 5 minutes. Samples were loaded onto 15% polyacrylamide SDS gels for separation of proteins and then transferred to nitrocellulose filters by semidry transfer (Biometra, Germany). The filters were blocked by 1% fat milk for 1 hour at room temperature and then incubated at room temperature, first with rabbit anti-human elastase/rabbit anti-human cathepsin G antibodies (Fitzgerald Industries International, USA) (diluted 1 : 1,000) for 2 hours, followed by incubation with goat anti rabbit-HRP conjugate (diluted 1 : 25,000) for an additional hour. The amount of the enzyme in each sample was calculated according to a positive control loaded on the same gel, active elastase (Sigma-Aldrich-cat. number E-8140)/active cathepsin G (Fitzgerald Industries International, USA).

The enzyme signal was detected on X-ray films using the chemiluminescence reagents of the EZ-ECL kit. The densities of the enzyme bands were determined by the BioCapt and Bio-Profile (Bio-1D) software.

### 2.6. Endothelial Cell Culture

Human umbilical vein endothelial cells (HUVEC) were cultured as described by Jaffe et al. [23], with minor modifications according to Lanir et al. [24]. Endothelial cell specificity (>95%) was confirmed by flow cytometry of cells stained with anti-human CD-34 PE-conjugated antibody (Becton Dickinson, USA) after cell detachment by trypsin/EDTA (Biological Industries, Beit Haemek, Israel).

### 2.7. Cocultivation of HUVEC with PMNLs

HUVEC were seeded in growth medium at a density of 1 × 10^5^ cells in six-well plates (Nalge Nunc International, USA) for 2 days under subconfluent conditions. Experiments were performed always between passages 2 to 4. In all experiments, prior to the addition of PMNLs, HUVEC were deprived of culture medium and kept in phosphate buffered saline (PBS) for 90 min at 37°C [25]. To prevent the direct contact of PMNLs with HUVEC, 0.45 *μ*m pore size cell culture inserts (25 mm tissue culture inserts, Nalge Nunc International) were placed into each well on top of HUVEC in direct contact with the medium, 1 mL of PBS [26]. PMNLs (10^6^) from NC or HD were seeded on these inserts and coincubated for 15 minutes at 37°C with HUVEC, as previously described [25].

### 2.8. Assessment of VE-Cadherin on HUVEC

*(1) By Flow Cytometry*. HUVEC, following 15 minute of cocultivation with PMNLs, were rinsed with PBS, scraped, centrifuged, resuspended, and incubated for 10 minutes with mouse anti VE-cadherin monoclonal antibody (United States Biological, MA, USA), followed by incubation with PE conjugated anti-mouse antibody (IQ products, The Netherlands). Irrelevant IgG-PE served as a nonspecific control of the fluorochrome (IQ products, The Netherlands).

*(2) By Immunohistochemical Staining*. HUVEC were grown on a chamber, a procedure that does not require cell transfer prior to visualization/staining, and cocultivated with PMNLs as previously described [25]. After cocultivation, the growth chamber was removed, and cells were fixed on the slides in 95% ethanol and stained with mouse anti VE-cadherin monoclonal antibody (United States Biological, MA, USA).

### 2.9. Evaluation of Soluble VE-Cadherin Fragments

*(1) In Plasma*. Soluble VE-cadherin was measured using western blot analysis. The plasma of NC subjects and HD patients was depleted of albumin and immunoglobulins. Following the addition of Laemli loading buffer containing 3% *β*-mercaptoethanol, the plasma was boiled at 100°C for 5 minutes. Samples were loaded onto 6% polyacrylamide SDS gels as described above. The whole 140 kDa molecule and the 100 kDa fragment were detected using mouse anti-human VE-cadherin (Cell Signaling Technology, MA, USA), while the 40 kDa fragment was detected using mouse anti-human VE-cadherin (United States Biological, Massachusetts, USA), which is not able to recognize the 140 kDa and the 100 kDa molecules. Thus, due to the different antibodies used, plasma sampled was loaded onto two gels, one stained for the evaluation of 100 + 140 kDa forms and one for the 40 kDa form. The levels of both whole and cleaved forms of VE-cadherin were assessed in HD plasma and compared with those of NC plasma. To calculate the levels of the enzymes, the bands were all compared to the same plasma sample run on all gels serving as a normalizing control.

*(2) In the Cocultivation Media*. The media of four cocultivation experiments were pooled in order to get detectable amount of these proteins. These media samples were treated with Vivaspin concentrators (Sartorius AG, Germany) for improved recovery of the low-concentration protein samples. Following the addition of Laemli loading buffer containing 3% *β*-mercaptoethanol, the plasma was boiled at 100°C for 5 minutes. Samples were loaded onto 6% polyacrylamide SDS gels as described above using mouse anti-human VE-cadherin (Cell Signaling Technology, MA, USA).

### 2.10. Statistical Analysis

Data is expressed as mean ± SD. In the boxes and whiskers presentations, the horizontal line in the middle shows the median (50th percentile), the top and bottom of the box show the 75th and 25th percentiles, respectively, and the whiskers show the maximum and the minimum values. The nonparametric Mann-Whitney test was used for comparing two independent groups. The two-paired Wilcoxon Signed Ranks test was used for comparing two dependent groups. Statistical significance was considered at  *P* < 0.05.

## 3. Results

### 3.1. PMNLs Priming in HD Patients

We reported previously that PMNLs from HD patients are primed [16]. To confirm these results we isolated PMNLs and measured the rate of superoxide release and the levels of surface CD11b, indices of PMNL priming [20]. The rate of superoxide release following PMA stimulation was higher in PMNLs isolated from HD patients than in those from NC (38.5 ± 3.9 versus 24.7 ± 5 nmoles/10^6^ cells/10 min, resp., *P* < 0.05). The membrane levels of CD11b were higher in PMNLs isolated from HD patients than in PMNLs from NC (49.9 ± 7.8 versus 32 ± 4 MFI resp., *P* < 0.05).

### 3.2. Elastase and Cathepsin G mRNA in PMNLs

Elastase and cathepsin G mRNA levels were not significantly different in NC versus HD PMNLs: 0.39 ± 0.14 versus 0.33 ± 0.22 relative density units, respectively, for elastase; *P* = ns; and 0.74 ± 0.1 versus 0.86 ± 0.47 relative density units, respectively, for cathepsin G; *P* = ns.

### 3.3. PMNL Intracellular Protein Levels of Elastase and Cathepsin G

**Figure 1:**
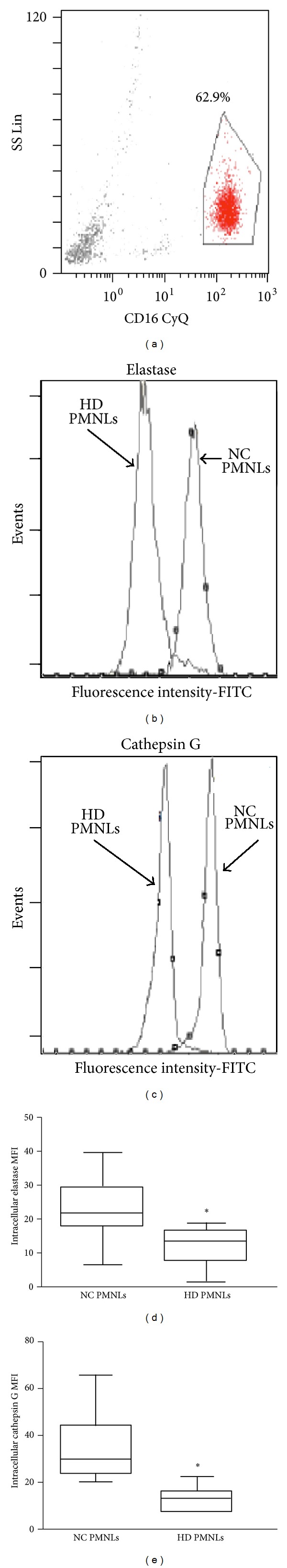
Intracellular levels of elastase and cathepsin G in PMNLs measured in whole blood. (a) Representative histogram of flow cytometry showing gating on the PMNL population which is CD16 positive cells. (b, c) Representative histogram of flow cytometry showing intracellular elastase and cathepsin G intensity in HD and NC PMNLs, respectively. (d, e) PMNL intracellular elastase and cathepsin G from NC subjects and HD patients detected by flow cytometry (*n* = 10). *P < 0.05 HD versus NC.

Flow cytometry measurements of elastase and cathepsin G in PMNLs measured in whole blood (Figures 1(a)–1(e)) showed higher levels of these enzymes in NC PMNLs than in HD PMNLs (average of 22.9 ± 2.7 versus 12.3 ± 1.7 MFI, resp., for elastase; *P* < 0.05 and 35 ± 5.2 versus 12.8 ± 1.4 MFI, resp., for cathepsin G; *P* < 0.05).

A significant negative correlation was found between the fluorescent intensity of intracellular elastase and cathepsin G and membrane CD11b expression on PMNLs, (*r* = −0.43 and *r* = −0.51, resp.; *P* < 0.05). This negative correlation indicates that the higher the priming the lower the amount of the intracellular enzymes.

### 3.4. Immunohistochemical Staining of Elastase and Cathepsin G in PMNLs

**Figure 2:**
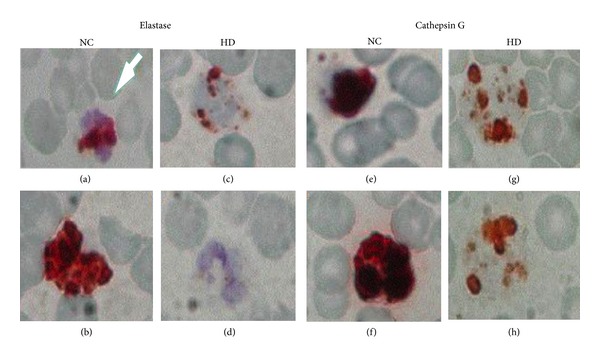
Localization of elastase and cathepsin G in PMNLs in whole blood smears. Indirect immunostaining of elastase (a–d) and cathepsin G (e–h) in blood smears of two NC subjects and two HD patients (light microscopy, magnification ×100).

The intracellular levels and locations of elastase and cathepsin G were also evaluated by immunohistochemical staining of PMNLs in smears of whole blood, as represented in Figure 2. Examination of the cells under a light microscope revealed that elastase and cathepsin G were abundant and distributed in PMNLs of NC, while sparse in HD PMNLs, and even missing in some patients.

### 3.5. Plasma Levels of Elastase and Cathepsin G

**Figure 3:**
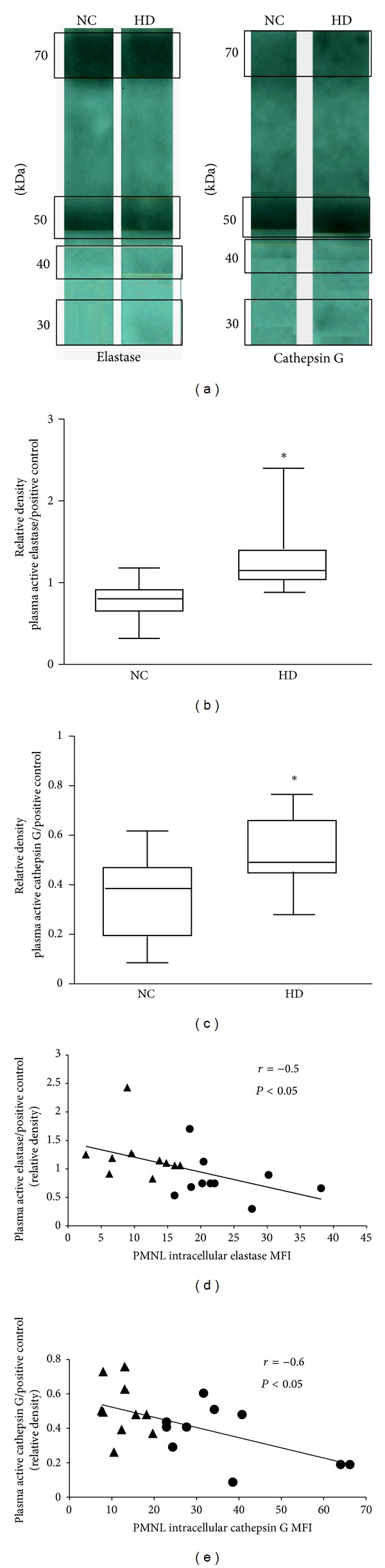
Levels of elastase and cathepsin G in HD and NC plasma. Proteins of plasma samples depleted from albumin and immunoglobulins of NC subjects and HD patient were separated on SDS-PAGE followed by western blot analysis. (a) A representative western blot of elastase and cathepsin G. Bands were visible at 30 kDa (inhibitor unbound) and higher M.W complexes of 40, 50, and 70 kDa in NC and HD plasma. ((b), (c)) Densities of unbound elastase and cathepsin G (30 kDa) from NC subjects and HD patients' plasma relative to commercial enzymes bands (*P > 0.05 HD versus NC; *n* = 10). (d) A negative correlation between plasma elastase levels of NC (●) and HD (▲) and the expression of their PMNL membrane CD11b measured in whole blood (*r* = −0.5; *P* < 0.05). (e) A negative correlation between plasma cathepsin G levels of NC (●) and HD (▲) and the expression of their PMNL membrane CD11b measured in whole blood (*r* = −0.6; *P* < 0.05).

Plasma of NC and HD was depleted of albumin and immunoglobulins in order to enrich it with elastase and cathepsin G. After depletion, plasma proteins were separated on SDS-PAGE followed by western blot analysis (Figure 3). HD plasma contained higher levels of free elastase and cathepsin G (30 KDa) than NC plasma (average of 1.3 ± 0.14 versus 0.76 ± 0.08 relative density units, resp., for plasma elastase; *P* < 0.05 and 0.52 ± 0.04 versus 0.34 ± 0.05 relative density units, resp., for plasma cathepsin G; *P* < 0.05) (Figures 3(b) and 3(c)). However, no significant differences in the levels of the higher molecular weight complexes (40, 50, and 70 Da) were found between NC and HD. A significant negative correlation was found between the amounts of the plasma levels of elastase and cathepsin G (30 KDa) and their levels in PMNLs (Figures 3(d) and 3(e)) (*r* = −0.5 and *r* = −0.6, resp.; *P* < 0.05).

### 3.6. Plasma Levels of Soluble VE-Cadherin Fragments

**Figure 4:**
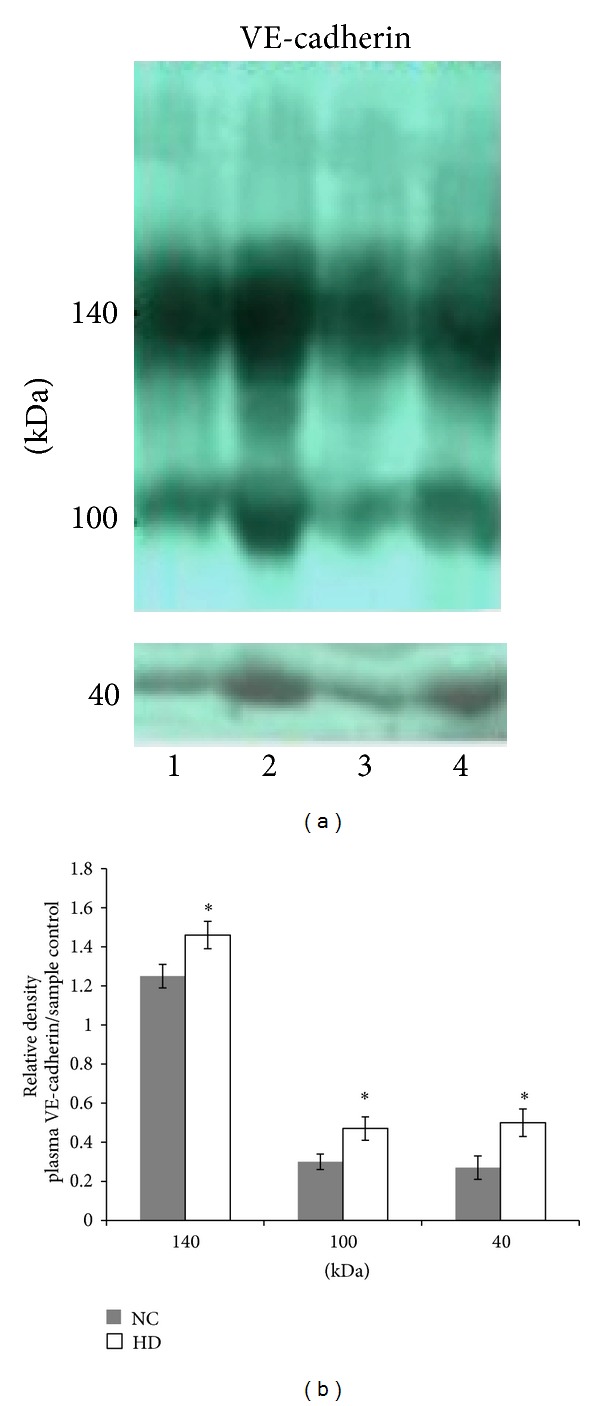
Soluble VE-cadherin fragments (140 and 100 kDa forms) in HD plasma versus NC plasma.Proteins of plasma samples depleted from albumin and immunoglobulins of NC subjects and HD patient were separated on SDS-PAGE followed by western blot analysis (a) Representative gels showing three sizes of VE-cadherin: the whole molecule: 140 kDa and the cleaved forms: 100 and 40 kDa in NC (1,3) and HD plasma (2,4). (b) Relative densities of VE-cadherin from NC subjects and HD patient plasma (*P > 0.05 HD versus NC; *n* = 10).

A significant difference in the levels of soluble VE-cadherin between NC and HD plasma was found: HD plasma contains higher levels of soluble VE-cadherin, both the whole molecule, 140 KDa (1.46 ± 0.07 versus 1.25 ± 0.06 relative density units; *P* < 0.05), and the cleaved form, 100 KDa (0.47 ± 0.06 versus 0.3 ± 0.04 relative density units; *P* < 0.05) (Figures 4(a) and 4(b)). Using anti VE-cadherin monoclonal antibody, we were able to detect the 40 kDa fragment of VE-cadherin in plasma. Again, HD plasma contains higher levels of 40 kDa VE-cadherin (0.5 ± 0.07 versus 0.27 ± 0.06 relative density units; *P* < 0.05) (Figures 4(a) and 4(b)).

**Figure 5:**
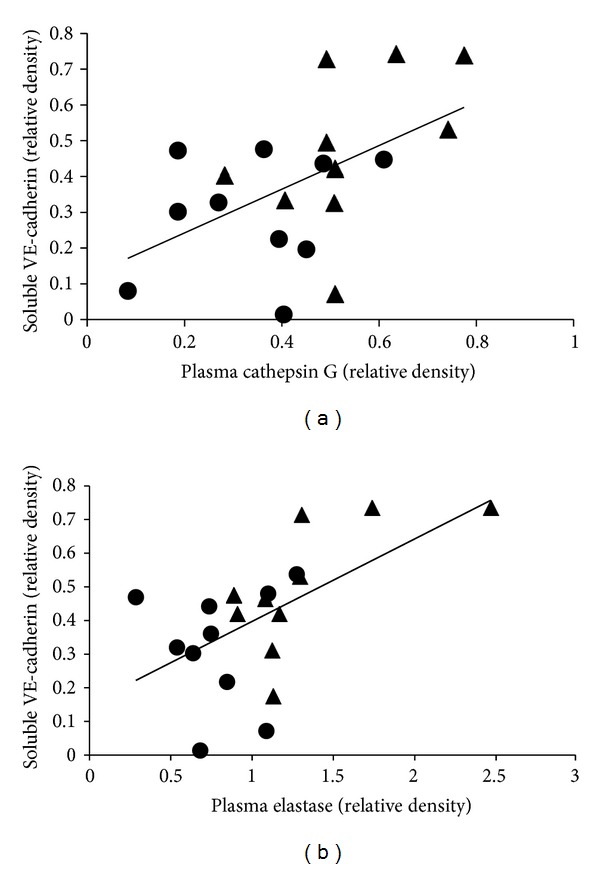
(a) Positive correlations between plasma active cathepsin G in plasma of NC (●) and HD patients (▲) and soluble VE-cadherin 100 kDa fragment (*r* = 0.51; *P* < 0.05; continuous line). (b) Positive correlations between plasma active elastase in plasma of NC and HD patients and VE-cadherin 100 kDa fragment (*r* = 0.53; *P* < 0.05; continuous line).

In addition, significant positive correlations were found between plasma active elastase and cathepsin G and the 100 KDa soluble VE-cadherin (Figures 5(a) and 5(b)) (*r* = 0.51 and *r* = 0.53 for elastase and cathepsin G, resp.; *P* < 0.05), suggesting that these proteins are involved in the degradation of VE-cadherin. No significant correlation was found between plasma active elastase and cathepsin G and the 140 and 40 KDa VE-cadherin forms.

### 3.7. HUVEC Membrane 40 kDa form of VE-Cadherin after Exposure to PMNLs

**Figure 6:**
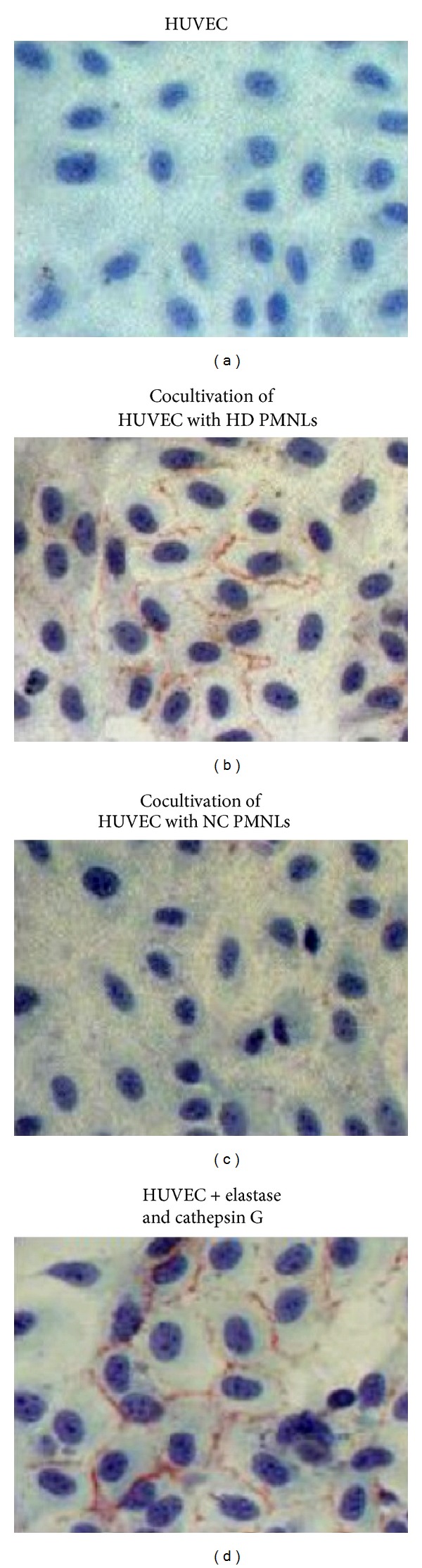
Indirect immunohistochemical staining of VE-cadherin (40 kDa form) in HUVEC after cocultivation with PMNLs and after exposure to elastase and cathepsin G (light microscopy, magnification ×20).

Control HUVEC and HUVEC exposed to NC PMNLs show light staining which indicates that the anti VE-cadherin did not bind to the VE-cadherin (Figure 6). However, when HUVEC were exposed to HD PMNLs, the staining was much intense, indicating strong binding of the antibody to VE-cadherin (Figure 6). When HUVEC were exposed only to proteases (0.02 U/mL elastase and 0.02 U/mL cathepsin G [12]), VE-cadherin staining was comparable to that after exposure to HD PMNLs. HUVEC membrane VE-cadherin was also determined by flow cytometry after exposure to HD and NC PMNLs. The level of VE-cadherin on unexposed HUVEC was 7.03 ± 3.84 MFI. Cocultivation of HUVEC with NC PMNLs resulted in a significant increase in VE-cadherin staining compared to untreated HUVEC (9.85 ± 5.77; *P* < 0.05). Cocultivation of HUVEC with HD PMNLs resulted in a greater increase in VE-cadherin staining (13.4 ± 7.57; *P* < 0.05).

### 3.8. Cocultivation Media Levels of Soluble 100 kDa Cleaved form of VE-Cadherin

**Figure 7:**
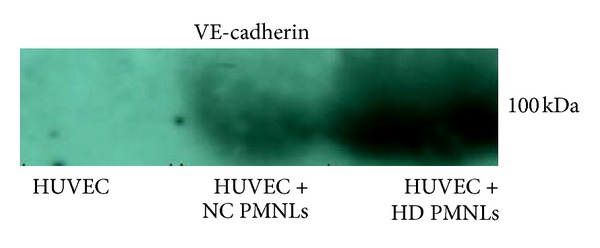
A representative gel showing soluble VE-cadherin (100 kDa form) in PMNLs and HUVEC cocultivation media separated by SDS-PAGE followed by western blot analysis.

We could not detect any soluble VE-cadherin in the control HUVEC which was not exposed to PMNLs. On the other hand, there was a detectable amount of soluble 100 kDa VE-cadherin in the cocultivation media when HUVEC were exposed to PMNLs (Figure 7). When HUVEC were exposed to HD PMNLs we detected 3 times more soluble VE-cadherin versus when exposed to NC PMNLs (Figure 7). We could not detect the 140 and 40 kDa forms of VE-cadherin in the media.

## 4. Discussion

The results of the present study demonstrate that the intracellular levels of elastase and cathepsin G in primed peripheral PMNLs of HD patients, before starting hemodialysis session, are significantly lower compared to healthy controls although their mRNA levels are similar. Lower intracellular levels of these proteases are associated with a significant increase in their plasma concentration, especially of the active form. This suggests that PMNL priming, common to HD patients, causes an increased release of these enzymes, resulting in their higher plasma levels in HD. Plasma from HD patients also contains higher levels of soluble VE-cadherin, in three molecular forms: 140 kDa, the whole molecule and two molecular fragments of this protein, 100 and 40 kDa.

We investigated the degree of PMNL priming of HD patients and the possible correlation between PMNL priming and the release of elastase and cathepsin G. First we supported our previous studies showing that PMNLs in HD patients are primed by using two different markers: elevated levels of CD11b and higher rate of superoxide release. Next, using immunocytochemical staining, immunoblotting, and FACS analyses we demonstrated that the staining intensities of the intracellular elastase and cathepsin G are reduced in the primed PMNLs of HD patients versus NC. A significant negative correlation was found between the intensity of intracellular elastase and cathepsin G and the degree of PMNL priming, as assessed by membrane CD11b expression, suggesting that the release of these proteolytic enzymes is greater when PMNLs are primed. Moreover, our findings refute the possibility that lower synthesis of these enzymes in the HD PMNLs caused their reduced intracellular levels, since the transcription levels of these enzymes were comparable in HD and healthy controls. This is in accordance with previous studies demonstrating that azurophilic granule proteins, such as these serine proteinases, are synthesized only at the promyelocyte and metamyelocyte stages, and remain stored in granules throughout terminal granulocytic differentiation [27, 28].

Since the intracellular protein levels of these enzymes were lower in HD, one would expect to find these enzymes in the surrounding milieu, the blood. As expected, significantly elevated levels of both proteolytic enzymes were found in blood of HD patients compared to NC.

The continuous release of these enzymes from PMNLs was already demonstrated during hemodialysis sessions [19], but to the best of our knowledge, a comparison with healthy subjects, especially as to the amounts of the active, noninhibitor bound enzymes, was not reported. Although the plasma levels of the higher molecular weights complexes of the enzymes (40, 50, and 70 kDa) were similar between plasma of HD and NC, the levels of the 30 kDa inhibitor unbound enzymes were significantly higher in HD plasma. Enzyme-inhibitor complexes are formed when elastase or cathepsin G binds to inhibitory proteins of the acute phase, such as serum proteins *α*1-antitrypsin (*α*1-AT) and *α*1-antichymotrypsin (*α*1-ACT) [10, 11]. The significant negative correlation between intracellular elastase and cathepsin G and the degree of PMNL priming and plasma levels of these enzymes further supports the enhanced degranulation of these primed PMNLs resulting in the release of the two proteolytic enzymes. This uncontrolled degranulation of primed PMNLs to the blood is potentially destructive in two ways: (1) reduced intracellular levels of these enzymes within the PMNLs decrease their capacity to neutralize and kill pathogens; (2) the continuous interactions of circulating HD PMNL with the blood vessel endothelial monolayer expose the vascular wall to chronic injury by these active circulating granular proteinases.

In the “response-to-injury” hypothesis suggested by Ross, atherosclerosis begins as a response to chronic minimal injury to the endothelium [29]. This injury leads to an array of endothelial cell responses, such as disruption of endothelial integrity, which will in the long run result in atherosclerosis [30]. Adherence junction (AJs) proteins, such as VE-cadherin, maintain endothelial integrity. Hermant at al. have shown that cleavage of VE-cadherin occurs following adhesion of fMLP-primed neutrophils to endothelial cell monolayer resulting in a soluble fragment of molecular mass of 38 KDa. Using specific inhibitors of neutrophil proteases, these researchers were able to identify elastase and cathepsin G as the major proteases involved in the cleavage of VE-cadherin. In addition, they demonstrated that purified elastase and cathepsin G are able to increase endothelial monolayer permeability* in vivo *[17]. Soeki et al. have shown that enhanced secretion of VE-cadherin from the arteries is associated with coronary atherosclerosis [31]. We imply that the elevated plasma levels of elastase and cathepsin G in HD patients, especially the active forms, cleave AJs proteins, such as VE-cadherin in the endothelium, an action associated with early steps in the atherosclerotic process.

This study demonstrates that HD plasma contains higher levels of soluble VE-cadherin present in three molecular forms: whole 140 kDa molecule and two fragments, the 100 and the 40 kDa forms. The 100 kDa protein is probably a product of the proteolysis of VE-cadherin by elastase and cathepsin G [17]. This assumption is supported by the significant positive correlation found between the amount of plasma active elastase and cathepsin G and the plasma levels of the 100 kDa fragment, the product of VE-cadherin degradation.

Our* in vitro* experiments showed that HD PMNLs can mediate cleavage of VE-cadherin from endothelial cells. This cleavage resulted in intense staining of the 40 kDa fragment on endothelial cells concomitantly with the appearance of the 100 kDa in the culture media. These results apparently conflict with our findings regarding low levels of elastase and cathepsin G found in HD PMNLs compared to controls. Yet, we propose that while in NC PMNLs these enzymes are stored within specific granules, HD PMNLs secrete their enzyme content to the surrounding milieu. Therefore, even trace amounts could elicit VE-cadherin cleavage as describe herein. The potential role of elastase and cathepsin G released from primed HD PMNLs in mediating VE-cadherin cleavage is further supported by our* in vitro* experiments where applying purified elastase and cathepsin G to HUVEC resulted in an increase staining of the 40 kDa fragment.

Our* in vitro* studies utilizing these proteinases on endothelial cells are in agreement with previous study demonstrating that after treatment with cathepsin G, endothelial cells became contracted and did not interact with neighbor cells. These effects depended on concentration and length of exposure to cathepsin G and ultimately cells became star-shaped until totally detached [26].

For conclusion, this study shows decreased elastase and cathepsin G expression in HD PMNLs while increased in HD plasma, most importantly in their active form. Furthermore, higher levels of soluble VE-cadherin were found in HD patient plasma. Thus, this study can provide a mechanism by which active elastase and cathepsin G are released from primed PMNLs, mediate VE-cadherin cleavage, and initiate endothelial dysfunction in these patients. Moreover, the implications of this study are beyond HD patients and can be implicated in all clinical states associated with primed PMNLs and accelerated atherosclerosis such as hypertension and diabetes.

## Figures and Tables

**Table 1:** Demographics of HD patients and NC subjects used in this study.

	Hemodialysis patients (*n* = 10)	Normal controls (*n* = 10)
Age (years)	54 ± 7	47 ± 5
Male/Female	5/5	5/5
Diabetes	3	—
Time on hemodialysis (months)	38 ± 6	—
